# Recent Advances in Molecular Mechanisms of Cancer Immunotherapy

**DOI:** 10.3390/cancers15102721

**Published:** 2023-05-11

**Authors:** Mateusz Kciuk, Esam Bashir Yahya, Montaha Mohamed Ibrahim Mohamed, Summya Rashid, Muhammad Omer Iqbal, Renata Kontek, Muhanad A. Abdulsamad, Abdulmutalib A. Allaq

**Affiliations:** 1 Department of Molecular Biotechnology and Genetics, University of Lodz, Banacha Street 12/16, 90-237 Lodz, Poland; 2 Doctoral School of Exact and Natural Sciences, University of Lodz, Banacha Street 12/16, 90-237 Lodz, Poland; 3 Bioprocess Technology Division, School of Industrial Technology, Universiti Sains Malaysia, Penang 11800, Malaysia; 4 Faculty of Applied Medical Sciences, Mohail Aseer, King Khalid University, Abha 62529, Saudi Arabia; 5 Department of Pharmacology & Toxicology, College of Pharmacy, Prince Sattam bin Abdulaziz University, Al-Kharj 11942, Saudi Arabia; 6 Shandong Provincial Key Laboratory of Glycoscience and Glycoengineering, School of Medicine and Pharmacy, Ocean University of China, Qingdao 266003, China; 7 Department of Molecular Biology, Faculty of Science, Sabratha University, Sabratha 00218, Libya; 8 Faculty of Applied Science, Universiti Teknologi MARA, Shah Alam 40450, Malaysia

**Keywords:** cancer immunotherapy, antitumor response, cytokines, immune checkpoints

## Abstract

**Simple Summary:**

The cancer-immunity cycle is characterized by various stimulatory and inhibitory factors, which together regulate the immune response and halt the extreme response that may lead to autoimmune disease. Immunotherapy of cancer has rejuvenated the field of tumor immunology and revolutionized treatment options. In the present review, we discussed the recent advances in different molecular mechanisms of cancer immunotherapy.

**Abstract:**

Cancer is among the current leading causes of death worldwide, despite the novel advances that have been made toward its treatment, it is still considered a major public health concern. Considering both the serious impact of cancer on public health and the significant side effects and complications of conventional therapeutic options, the current strategies towards targeted cancer therapy must be enhanced to avoid undesired toxicity. Cancer immunotherapy has become preferable among researchers in recent years compared to conventional therapeutic options, such as chemotherapy, surgery, and radiotherapy. The understanding of how to control immune checkpoints, develop therapeutic cancer vaccines, genetically modify immune cells as well as enhance the activation of antitumor immune response led to the development of novel cancer treatments. In this review, we address recent advances in cancer immunotherapy molecular mechanisms. Different immunotherapeutic approaches are critically discussed, focusing on the challenges, potential risks, and prospects involving their use.

## 1. Introduction

**Figure 1:**
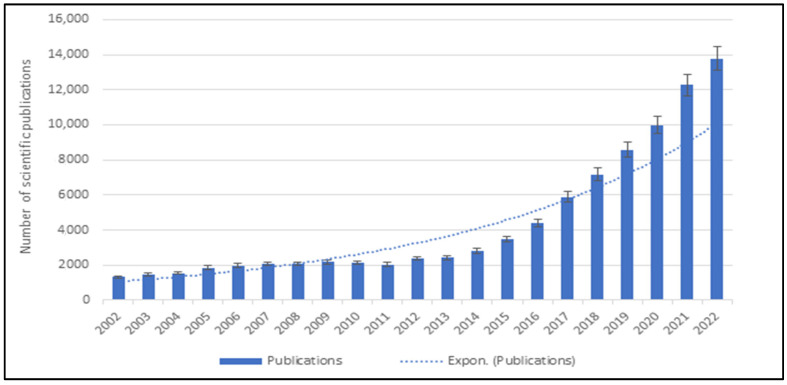
The number of scientific publications related to cancer immunotherapy (Search done through Science Direct database on 30 March 2023).

The therapeutic options for cancer treatment have been constantly evolving, resulting in the prolongation of life expectancy among many cancer patients [1]. Nonetheless, cancer is still among the leading causes of death worldwide and is considered a major public health concern [2]. The lack of effective cancer treatments capable of overcoming the natural acquisition of tumor resistance is the main reason for high mortality among cancer patients. Conventional therapies such as surgery, chemotherapy, radiotherapy, or hormone therapy are either not available or highly toxic for patients [3]. The recent breakthroughs in the development of new generations of cancer immunotherapies and understanding of tumor immune biology have opened a brand new era of the war against cancer. Immunotherapy aims to improve the natural immune responses directed against tumor cells with fewer off-target effects compared to the generalized chemotherapies and other agents designed to directly kill cancer cells [4,5]. Thus, immunotherapy has been recognized in the scientific community and is now considered a promising approach for the treatment and even cure of various types of tumors. A significant acceleration in the number of scientific publications each year confirms the promising potential of immunotherapy in cancer treatment. Figure 1 present the number of publications in the Science Direct database (https://www.sciencedirect.com/, accessed on 30 March 2023) related to cancer immunotherapy in the past two decades.

Cancer immunotherapy focuses on shifting the target from tumor cells themselves to the patient’s immune system to enable its mobilization and to enhance the activation of the antitumor immune response. This helps the immune cells recognize, attack, and eventually eliminate the tumor cells [6]. The interest in cancer immunotherapy has gained increasing attention in recent years compared to the other treatment options. The discovery of different cancer immune checkpoints, the extensive understanding of the role of cytokines in the activation of antitumor response, advances in genetic engineering technology, and the generation of genetically modified immune cells changed the landscape of cancer treatment and made the promise of the final cure for this disease nearer than ever. Numerous review articles have been published recently discussing the concept of immunotherapy [7], brain tumors immunotherapy [8], and the role of certain immune cells in cancer therapy such as macrophages [9] and dendritic cells (DCs) [10]. Fukumura et al. [11] discussed the opportunities and challenges of enhancing cancer immunotherapy using antiangiogenics, without referring to the types and different mechanisms of cancer immunotherapy. Riley et al. [5] further explained delivery technologies in cancer immunotherapy.

The current review extensively covers the chronological development of cancer immunotherapy, from the first attempts to develop a cancer vaccine to the current state, going through the natural immune response and immune surveillance. Different techniques of cancer immunotherapy will be furtherly discussed including regulation of immune checkpoints and their inhibitors, oncolytic virus therapies, cancer vaccines, cytokine therapies, and adoptive cell transfer. Finally, the challenges regarding immunotherapy and potential risks as well as future directions are addressed in the present review.

## 2. Cancer and Immunotherapy

### 2.1. Incidence, Progression, and Metastasis of Cancer

**Figure 2:**
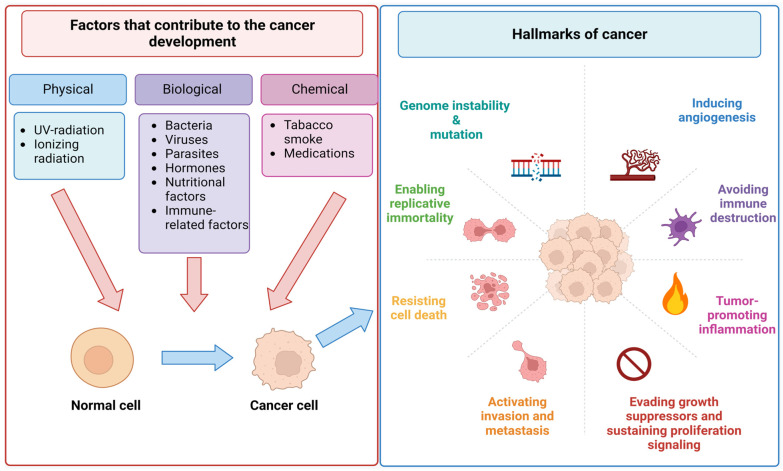
The main causes of human cancers and hallmarks of cancer established by Hanahan and Weinberg. Created with BioRender.com, accessed on 23 April 2023.

For decades, scientists have been extensively studying the biological nature of cancer cells to uncover the basis of the origin of human cancers [12]. Many studies have confirmed the causative roles of chemical (tobacco smoke, medications), physical (UV-radiation, ionizing radiation), and biological (bacteria, viruses, parasites, etc.) carcinogens as well as genetic and epigenetic abnormalities (Figure 2) [13,14,15].

The development of tumors from single cells that start to proliferate in an aberrant manner, also known as tumor clonality is among the fundamental characteristics of cancer. However, just because a tumor has a clonal origin does not mean that the precursor cell from which it originated had already acquired the other characteristic of malignant cells. Cancer development is a multistep process characterized by unrestricted cell proliferation, invasion, and metastasis resulting from the accumulation of genetic and epigenetic alterations. Furthermore, cancer cells release signaling molecules to support the formation of a network of capillaries and blood vessels, to provide nutrients and energy for the growing tumor, or to facilitate migration from the site of the origin to the distant locations of the body [16]. These features accompanied by avoidance of immune destruction and generation of tumor-promoting inflammation constitute the hallmarks of cancer introduced by Hanahan and Weinberg in 2000 [17,18,19,20]. However, even after the successful treatment or removal of tumor, circulating tumor cells (metastatic cancer cells) have been observed in the blood circulation of some individuals not presenting any symptoms of cancer and suggesting that these individuals may still harbor dormant cancer cells in some tissues of their body [21]. Furthermore, the development of malignant lesions from normal cells requires a long-term dormancy time, even after direct exposure to known carcinogens [22,23]. Metastatic dormancy is a late stage in cancer progression that occurs after cell extravasation at a secondary site, where metastatic cells cease proliferation but remain dormant. When the microenvironment is favorable, they re-initiate proliferation and colonization, sometimes years after the original tumor has been treated [21].

The tumor microenvironment is a complex ecosystem that consists of various cells and non-cellular components surrounding a tumor. These cells play critical roles in tumor growth, invasion, and response to therapy [24]. Cancer cells, which are the primary cells within the tumor itself. They have acquired genetic and epigenetic alterations that allow them to proliferate uncontrollably, invade surrounding tissues, and evade the immune system. Cancer cells secrete various factors that influence the behavior of other cells in the microenvironment [25]. Cancer-associated fibroblasts (CAFs) are a type of activated fibroblast that are commonly found in the tumor stroma. They produce extracellular matrix (ECM) proteins, growth factors, and cytokines that promote tumor growth, angiogenesis (formation of new blood vessels), and invasion. CAFs can also remodel the ECM, creating a physical barrier that limits immune cell infiltration into the tumor [26]. Various immune cells also infiltrate the tumor microenvironment, either attracted by signals released by the tumor or recruited by other immune cells. These include; Tumor-infiltrating lymphocytes are a diverse population of T cells that can include both cytotoxic CD8+ T cells, which directly kill cancer cells, and regulatory T cells (Tregs), which suppress immune responses. The balance between effector T cells and Tregs influences the immune response within the tumor [27]. Tumor-associated macrophages (TAMs) are a type of immune cell derived from circulating monocytes. They can have different functional phenotypes, including M1-like (pro-inflammatory) or M2-like (immunosuppressive). TAMs play roles in promoting angiogenesis, tissue remodeling, and suppressing immune responses. Other cells have also been reported to have critical role including dendritic cells, endothelial cells, mesenchymal stem cells and other stromal cells. The tumor microenvironment may also contain other stromal cells, such as adipocytes and pericytes, which can influence tumor behavior through various mechanisms, including the secretion of factors that promote tumor growth and invasion [28]. It is important to note that the composition and function of the tumor microenvironment can vary depending on the type of cancer and the stage of the disease. The interactions between these different cell types within the tumor microenvironment are highly complex and can have both promoting and inhibitory effects on tumor progression and response to therapy. Understanding these interactions is crucial for the development of effective cancer treatments.

### 2.2. Antitumor Natural Immune Response

**Figure 3:**
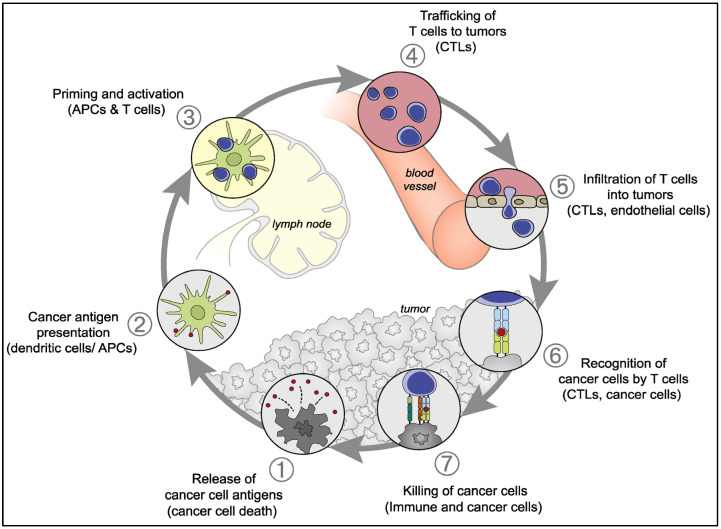
Schematic illustration of the cancer-immunity cycle. (CTL: cytotoxic t lymphocytes; APC: an antigen-presenting cell). Adapted with permission from Chen et al. [40].

Based on current knowledge of cancer immune response, three separate steps have been described that must be completed to generate effective antitumor immunity. Antitumor immunity initiates when DCs recognize and sample cancer antigens [29]. The DCs must receive a suitable activation signal for their maturation, to allow them to further differentiate [30]. Tumor cells have been reported to overexpress certain endoplasmic reticulum stress proteins including calreticulin (CRT) on their cell surface [31]. These proteins seem to promote their phagocytosis by the activated DCs, enabling their presentation on class I and class II molecules of major histocompatibility complex (MHC) [32]. Following antigen presentation, DCs migrate to the nearest lymphoid organs and generate protective T-cell responses depending on many factors such as the interaction between T-cell co-stimulatory molecules and DC surface receptors, DC maturation stimuli, and the type of presented antigens [33]. The interaction of the surface receptors: T-cell-specific surface glycoprotein CD28 (CD28) on T cells with CD80/86 present on antigen-presenting cells (APCs) such as DCs will lead to the promotion of antitumor T-cell response. However, other receptors on T cells such as cytotoxic T-lymphocyte protein 4 (CTLA4) may interact with CD80/86 and suppress the T-cell activation, proliferation, and cytokine production. Similarly, the interaction between the programmed cell death protein 1 (PD-1) receptor expressed on the surface of T cells and programmed cell death ligand 1 (PD-L1) expressed on the surface of APCs, tumor cells, and other cells will lead to the suppression of T-cell responses and will promote the formation of regulatory T cells (T regs) [32]. Calcium (Ca^2+^) signaling is of paramount importance to immunity. Increasing intracellular calcium ([Ca^2+^]i) levels play a pivotal role in the activation of the immune system [34]. Calcium signaling is a fundamental mechanism that regulates various aspects of immune cell function, including cell activation, proliferation, differentiation, and effector responses [35,36,37]. In a recent study, the authors found two mechanisms that may account for the Ca^2+^ optimum of cancer cell killing—migration velocity and persistence at a moderate optimum between 500 and 1000 μm [Ca^2+^]o in CTLs, and lytic granule release at the immune synapse between CTLs and cancer cells is increased at 146 μm compared to 3 or 800 μm [35]. Activated T cells will leave the lymph node and migrate to the tumor bed. The exact type of activated T-cell response varies, but many studies suggested the generation of cytotoxic T cells or CD8+ effector T cells, which possess cytotoxic potential and kill cancer cells [38,39]. Figure 3 presents the steps of cancer immunity.

A balanced and highly controlled cancer-immunity cycle, characterized by various stimulatory and inhibitory factors, regulates the immune response and halts the extreme response that may trigger an autoimmune reaction. DCs have been reported to stimulate antibody-mediated as well as natural killer (NK) cell responses, which contribute to antitumor activity [41]. DCs are thought to be the link between innate and adaptive immunity because they can sample antigenic information released by cancer and present it to naive CD8+ and CD4+ T cells in draining lymph nodes via MHC-I and MHC-II molecules [42]. Many mechanisms have been proposed for tumor cells to avoid the immune response when cancer-specific T cells enter the tumor bed. These include the production of various surface molecules that suppress T-cell receptor signaling (CTLA-4 or PD-L1), localized accumulation of Treg cells, or down-regulation of MHC-I expression on the tumor cell’s surface [43]. The expression of such suppressive ligands has been linked to oncogenic mutations found in many malignancies (e.g., phosphatidylinositol 3,4,5-trisphosphate 3-phosphatase and dual-specificity protein phosphatase (PTEN) loss). Tumors can also produce immunosuppressive molecules such as indoleamine 2,3-dioxygenase (IDO), which limits tryptophan accessibility and restricts T-cell activity. Finally, stroma cells of the tumor can also hamper T-cell activity by blocking their adhesion and preventing the colonization of tumor cells in distant sites [44].

As a result, the success of cancer immunotherapy relies on its ability to overcome all of these major obstacles. Moreover, tumor-associated antigens are identical to self-antigens because they are part of the host’s tissues, making their use extremely difficult. Otherwise, the treatment could trigger autoimmune responses dangerous for patients. However, as will be discussed later, there appears to be a path to clinical success for cancer immunotherapy via the development of numerous novel approaches.

### 2.3. Chronological Development of Cancer Immunotherapy

Following various microbial infections, spontaneous tumor regression has been observed inspiring the first immunotherapeutic approach [45]. W. B. Coley found in the 1800s that inducing fever aids the immune system’s fight against the tumor, resulting in its regression. Coley’s approach to tumor therapy at the time, and even now, represents a once-in-a-lifetime opportunity to develop a safe, relatively inexpensive, and yet effective cancer treatment [46]. A few years later, the events regarding cancer immunotherapy have steadily developed as presented in Table 1.

## 3. Mechanisms of Cancer Immunotherapy

**Figure 4:**
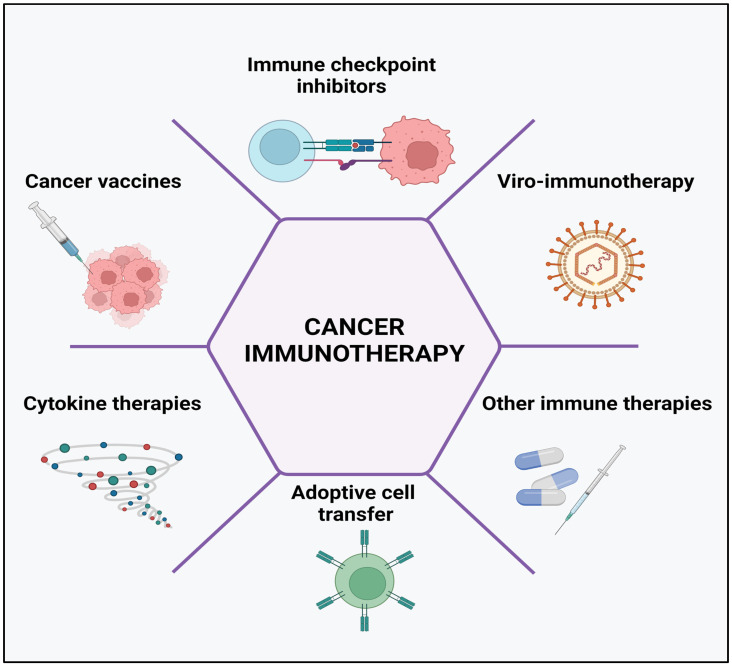
Cancer immunotherapy types include the use of immune-checkpoint inhibitors, cancer vaccines, cytokines, viruses, and adoptive cell transfer. Created with BioRender.com, accessed on 23 April 2023.

The human immune system comprises various defense mechanisms against all potential threats including arising cancer cells. Immune surveillance continuously recognizes and eliminates any transformed tumor cells through numerous mechanisms [75]. Cancer immunotherapy has been described as the fourth pillar of therapy against the tumor, which may surpass the effectiveness of conventional therapies such as surgery, radiotherapy, and chemotherapy [76]. Particularly, it has been listed by Science in 2013 among the top ten annual significantly valuable scientific breakthroughs [77]. Zhang et al. [78] classified the mechanisms of cancer immune therapies into five major groups (Figure 4)—the regulation of immune checkpoints, oncolytic virus therapies, cancer vaccines, cytokine therapies, and adoptive cell transfer.

### 3.1. Regulation of Immune Checkpoints

Cancer therapy by regulation of immune checkpoints is an approach that aims to activate the immune system against cancer cells by targeting proteins that act as negative regulators of immune responses. Cancer cells can evade immune surveillance by expressing multiple immune-checkpoint proteins, such as programmed cell death ligand 1 (PD-L1), CD80/86, and Gal9, which inhibit T-cell activation and proliferation [79,80]. Therapies that target these immune-checkpoint proteins, such as immune-checkpoint inhibitors, have shown remarkable success in the treatment of various types of cancer [81,82,83]. These drugs block the interaction between immune-checkpoint proteins and their ligands, thereby releasing the inhibition of T-cell function.

PD-1 is an immunoglobulin (Ig) class protein that is expressed in T, B, myeloid, and NK cells. It has been demonstrated that PD-1 has two primary interacting partners: PD-L1 and PD-L2. PD-L1 is expressed by the APCs, hematopoietic and non-hematopoietic cells, whereas PD-L2 is expressed by activated macrophages and DCs. When PD-1 interacts with its ligands on other T cells, T-cell activation is hindered. The efficacy of TCR signaling is correlated with PD-1 expression in T cells. Its normal physiological function is to prevent the overactivation of T cells in the absence of the antigen. However, chronic viral infection and malignancy can trigger a constitutive PD-1/PD-L1 cell surface expression, which in turn inhibits the immune response and impairs T-cell function. This topic was reviewed by other authors [84,85,86,87,88] and the promises and limitations of the PD-1/PD-L1 blockage were recently described by our group and will not be discussed in detail [89].

CTLA-4 is a protein receptor found on the surface of T cells (CD4+ and CD8+). It is essential for immune response control, particularly in the context of T-cell activation and tolerance. CTLA-4 has a similar structure to CD28, another protein receptor produced on T cells that plays an important role in T-cell activation. CTLA-4, on the other hand, offers an inhibitory signal that reduces T-cell activation and proliferation, whilst CD28 gives a co-stimulatory signal that increases T-cell activation. CTLA-4 competes with CD28 for binding to APCs co-stimulatory molecules CD80 (B7-1) and CD86 (B7-2). When CD28 attaches to these molecules, it provides a stimulating signal, whereas CTLA-4 delivers a negative signal, limiting T-cell activation and proliferation [90,91,92].

Despite both of these molecules contribute to the inhibition of anticancer immunity, the timing of down-regulation, the underlying signaling mechanisms, and the anatomic locations of immunological suppression by these two immune checkpoints are different. PD-1 is more widely expressed on activated T cells, B cells, and myeloid cells than CTLA-4, which is only found on T cells. While CTLA-4 is active during the T-cell activation’s priming phase, PD-1 is active during the T-cell activation’s effector phase, largely within peripheral tissues. These characteristics suggest that anti-CTLA-4 and anti-PD-1 treatments may have cumulative and synergistic benefits in the treatment of the disease [93,94,95]. PD-1 inhibitors, such as pembrolizumab and nivolumab, have been approved for the treatment of melanoma, non-small-cell lung cancer, bladder cancer, and other types of cancer. CTLA-4 inhibitors, such as ipilimumab, have been approved for the treatment of melanoma, and combinations of PD-1 and CTLA-4 inhibitors are being investigated for the treatment of several types of cancer. Other immune-checkpoint inhibitors, such as those targeting T-cell immunoglobulin and mucin-domain containing-3 (TIM-3), are currently under investigation in clinical trials [96,97].

**Figure 5:**
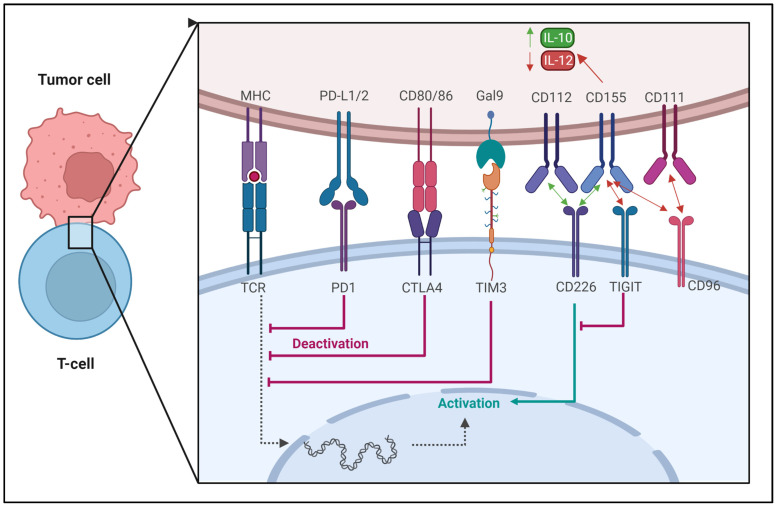
Schematic illustration of the mutual regulation by various immune checkpoints between cancer cells and T cells. The interaction between Major Histocompatibility Complex class (MHC) molecules and the T-cell receptor (TCR) is a critical step in the activation of T cells. The interaction between PD-1 (programmed cell death protein 1) on the surface of T cells and PD-L1/2 (programmed cell death ligand 1/2) expressed on tumor cells inhibits TCR signaling. A similar effect is observed when CD80/CD86 molecules present in cancer cells bind with cytotoxic T-lymphocyte-associated protein 4 (CTLA4). Inhibitory effects on TCR signaling and T-cell activity have been attributed to the interaction between TIM-3 (T-cell immunoglobulin and mucin-domain containing-3) and its ligand Galectin-9 (Gal-9). In turn, T-cell immunoreceptor with Ig and ITIM domains (TIGIT) and CD96 can suppress T-cell activation by binding to CD155 with a higher affinity than CD226, which in turn outcompetes CD226 for the binding site. The binding of TIGIT with CD155 induces an inhibitory signal within tumor cells. This signal up-regulates IL-10 while simultaneously down-regulating IL-12. TIGIT hinders CD226 and CD155 from engaging in interaction by disrupting the homodimerization of CD226. It is currently unknown whether or not TIGIT can directly provide co-inhibitory signals in T cells once it has bound to CD155. CD226 is responsible for delivering co-stimulatory signals by binding to CD112 and CD155. CD96 is responsible for delivering co-inhibitory signals when it binds to CD111 and CD155. Created with BioRender.com, accessed on 23 April 2023. Based on [76].

TIM-3 is another cell surface immune-checkpoint molecule expressed in immunological cells such as T cells, Tregs, DCs, B cells, macrophages, NK cells, and mast cells. TIM-3 binds to four different ligands: galectin-9 (Gal-9), CEACAM-1 (carcinoembryonic antigen cell adhesion molecule 1), HMGB1 (high-mobility group protein B1), and PS (phosphatidylserine). TIM-3 mediates T-cell exhaustion, which suppresses antitumor immunity. The selective blockage of TIM-3 enhances anticancer immunity through increased IFN-γ production by T cells. TIM-3 inhibitors have been studied in animal models and clinical trials [98,99,100,101]. The blockade of a single checkpoint in many cases may not achieve the desired therapeutic effect [102,103]. Figure 5 presents the mutual regulation by various immune checkpoints between cancer cells and T cells.

TIGIT (T-cell immunoreceptor with Ig and ITIM domains) is a protein that is expressed on various types of immune cells, including T cells and NK cells. TIGIT is up-regulated on activated T cells, particularly on Tregs, follicular helper T cells (Tfh), and memory T cells [104]. TIGIT is a co-inhibitory receptor that interacts with its ligands, CD155 (poliovirus receptor) and CD112 (nectin-2), which are expressed on APCs and some tumor cells. The binding of TIGIT to its ligands leads to the inhibition of T-cell activation and proliferation, as well as the suppression of cytokine production. Therefore, TIGIT plays an important role in the regulation of the immune response and maintenance of immune homeostasis. Targeting TIGIT has emerged as a potential immunotherapy strategy for cancer and autoimmune diseases [105]. On the other hand, CD96 (also known as TACTILE) is a protein that is primarily expressed on activated T cells, including CD8+ T cells, Tregs, NKT cells, and some subsets of memory T cells. CD96 is also expressed in some tumor cells [106]. CD96 interacts with its ligand, CD155, which is widely expressed in various cell types, including T cells, DCs, tumor cells, and many other cell types. CD96 has been shown to play a role in T-cell activation and effector functions, as well as in the regulation of immune tolerance and autoimmunity. CD226 (also known as DNAM-1) and CD112 (nectin-2) are also cell surface receptors that are expressed on various immune cells, including T cells, NK cells, and DCs, as well as on many tumor cells. CD226 interacts with its ligands, CD155 and CD112, and plays a role in the activation and cytotoxicity of NK cells and CD8+ T cells [107,108]. TIGIT and CD96 are more likely to bind and suppress T-cell activation than CD226, which has a lower affinity for CD155. TIGIT binding to CD155 on the tumor cells leads to the up-regulation of IL-10 and down-regulation of IL-12 [106].

**Figure 6:**
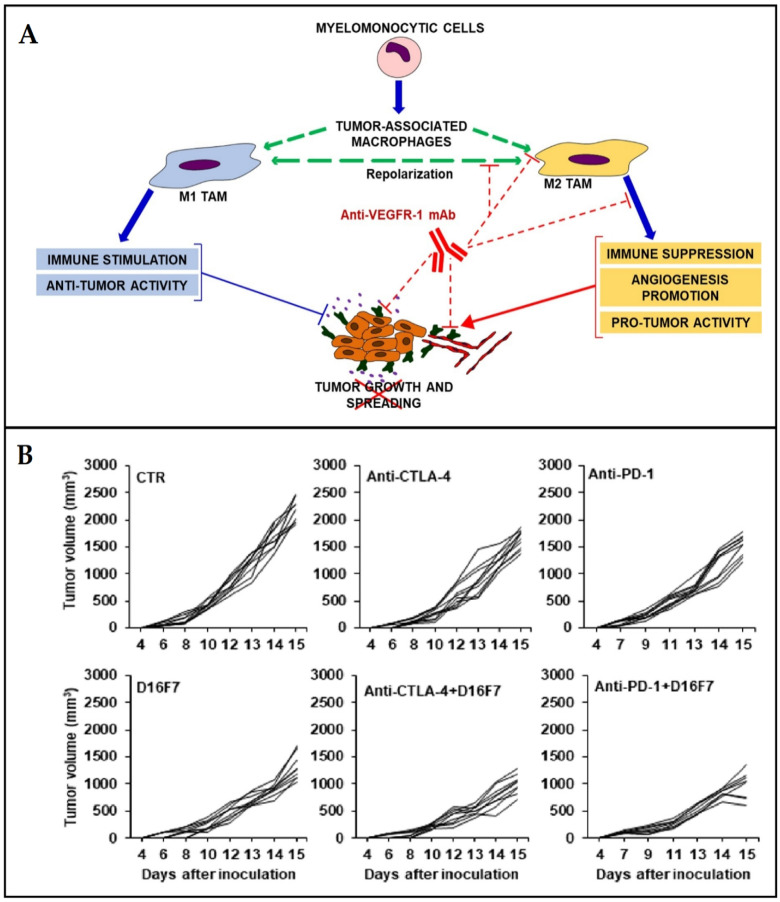
Vascular endothelial growth factor receptor-1 (VEGFR-1)-mediated approach for improving antitumor immune response. (**A**) Schematic illustration of the principal; (**B**) antitumor activity of the anti-VEGFR-1 monoclonal antibody (mAb D16F7) in combination with anti-CTLA-4 or anti-PD-1 mAbs. Adapted with permission from Lacal et al. [110].

Vascular endothelial growth factor receptor-1 (VEGFR-1) is a crucial receptor that plays a major role in tumor progression as well as in the resistance to treatment methods based on immune-checkpoint inhibitors [109]. Activation of VEGFR is involved in myeloid progenitors mobilization from the bone marrow which later infiltrates the tumor. Lacal et al. [110] recently developed an anti-VEGFR monoclonal antibody (anti-VEGFR-1 mAb) able to inhibit the growth and proliferation of melanoma in preclinical in vivo models. In addition, the reduction in macrophage progenitor mobilization and tumor cell infiltration by myeloid cells was observed. The same authors indicated significant up-regulation in the expression of VEGFR-1 in human-activated macrophages 2 (M2) compared to activated macrophage 1 (M1) cells. The exposure to the designed monoclonal antibody (D16F7 mAb) decreases the chemotaxis of macrophages in vitro. The results of in vivo treatment in melanoma-bearing mice resulted in significant inhibition of tumor growth due to the alterations in the tumor microenvironment, which lead to a decrease in melanoma infiltration by M2 as well as PD-1+ and FoxP3+ cells. These modifications resulted in an increase in M1/M2 and CD8+/FoxP3+ cell ratios in favor of antitumor immune response [110]. Figure 6 presents the mechanism of using VEGFR-mediated signaling inhibition for the reduction in tumor infiltration by improving antitumor immune response and enhancing the efficacy of immunotherapy using immune-checkpoint inhibitors. Studies by Chauvin et al. [111] revealed the overexpression of PD-1 and TIGIT on most tumor-infiltrating CD8+ T cells and tumor antigen-specific circulating CD8+ T cells. Combined blockade of both checkpoints (TIGIT and PD-1) has shown significant enhancement in the antitumor function of cytotoxic CD8+ T cells compared to blocking PD-1 alone [112], this dual blockade seems to depend on CD226 signal transduction [113]. The combination of specific checkpoint blockade can be used to improve immune response and constitutes a promising direction for cancer immunotherapy.

Immune-checkpoint inhibition can be used to treat various malignancies including melanoma, lung cancer, bladder cancer, kidney cancer, and Hodgkin’s lymphoma. Unlike standard cancer therapies such as chemotherapy, immune-checkpoint inhibition can result in long-term remission and even tumor eradication in some individuals. Because they exclusively target the immune system rather than all rapidly dividing cells in the body, immune-checkpoint inhibitors have fewer side effects than standard chemotherapy. Despite increased safety profile, they can still induce substantial consequences such as lung, liver, and other organ inflammation. Additionally, the inhibition success rate depends on the type of cancer and other factors such as the patient’s immune system status. Additionally, certain cancer cells may acquire resistance to immune-checkpoint inhibitors, resulting in therapy failure. Because immune-checkpoint inhibitors are a relatively new type of cancer treatment, long-term data on their efficacy and potential side effects is also lacking. This topic was previously described by other authors [114,115,116,117].

### 3.2. Viro-Immunotherapy

Viruses have been widely investigated for being the ideal therapeutic option for various types of cancers in combination with immune-checkpoint inhibitors and many other types of immunotherapeutic approaches [118,119]. Oncolytic virotherapy focuses on the development of highly specific genetically engineered viruses for targeting only cancer cells. Following the recognition of cancer cells expressing the particular molecules, these viruses infect and kill cancer cells through their lysis. Owing to their ability to accommodate gene insertions, oncolytic viruses have been armed with transgenes to produce immune-stimulatory signals to improve antitumor immune response [120,121]. Numerous clinical and preclinical studies on the use of different oncolytic viruses in cancer immunotherapy have been conducted and reported promising antitumor potential. Table 2 presents the most commonly used oncolytic viruses for viro-immunotherapy.

**Figure 7:**
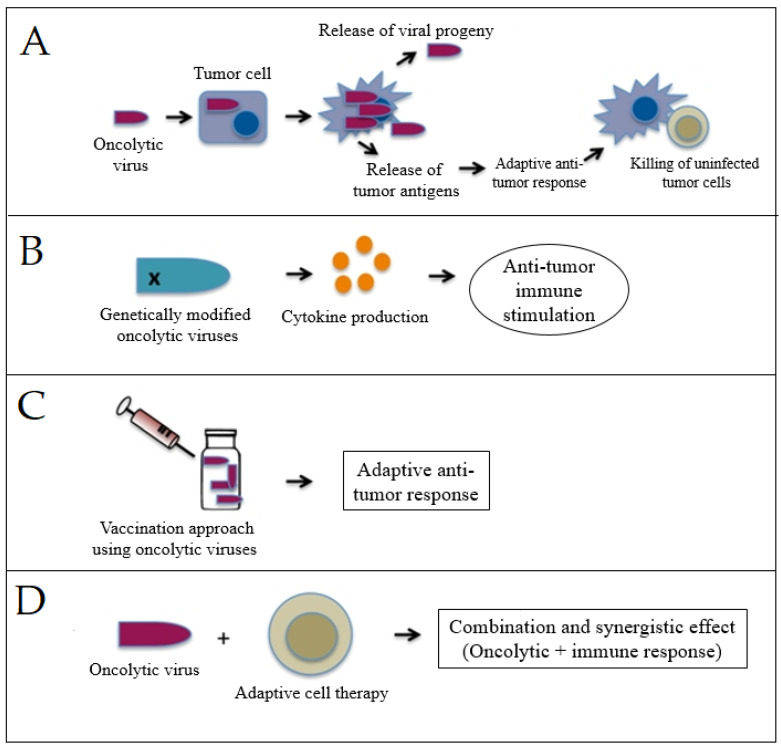
Schematic representation of oncolytic viro-immunotherapy strategies: (**A**) modification of endogenous genes of the virus for stimulating interferon induction, (**B**) virus-mediated expression of immune-stimulatory cytokines, (**C**) oncolytic cancer vaccine approach, and (**D**) synergistic effect of adaptive cell therapy and oncolytic viruses. Adapted with permission from Melzer et al. [130].

Vesicular stomatitis viruses (VSV) have been reported to have several beneficial properties that favor their use for immunotherapy, including inherent tumor specificity, rapid replication kinetics, and the capacity to elicit a wide variety of immune responses [129]. Melzer et al. [130] summarized the strategies of this novel platform for the enhancement of the immune-stimulating potential as presented in Figure 7. Modification of the virus’s endogenous genes to stimulate more interferon induction and virus-mediated expression of immune-stimulatory cytokines are two strategies that have been widely used with VSVs-based viro-immunotherapy or employed with other viruses to boost antitumor immune responses [131,132]. Vaccination approaches based on viruses have also been used to stimulate and even improve adaptive immune responses against particular tumor antigens [133].

In comparison to chemotherapies, oncolytic viro-immunotherapy has many advantages [134,135] including (a) selective targeting of cancer cells. Cancer cells can be specifically targeted and destroyed with viro-immunotherapy while healthy cells are unharmed. This is because the viruses have been engineered to specifically target cancer cells, reducing the likelihood of unintended effects. (b) Induction of immunogenic cell death: Immunogenic cell death triggered by oncolytic viruses primes the immune system to target and destroy cancer cells. As a result, the immune system has an increased likelihood of killing cancer cells even if they have metastasized. (c) Synergistic effect with other therapies: Combining viro-immunotherapy with standard cancer therapies such as chemotherapy and radiation has been shown to improve patient outcomes. Some clinical and preclinical research indicates that combination therapy is more effective than either medication alone. (d) Minimal systemic toxicity: As the viruses used in viro-immunotherapy are engineered to specifically target and destroy cancer cells, they are found to have low systemic toxicity. This lessens the likelihood of side effects that are usually associated with conventional anticancer therapies. (e) Potential to overcome drug resistance: Drug resistance is a serious problem impacting the usefulness of cancer therapies. Oncolytic viruses can specifically target and kill cancer cells that have developed resistance to other therapies. (f) Ability to target cancer stem cells: Cancer stem cells are a subpopulation of cancer cells that are believed to be responsible for tumor initiation, upkeep, and resurgence. Viro-immunotherapy may be able to target these cells. Treatment outcomes may improve and last longer if cancer stem cells are targeted [121,136,137,138]. However, their use can be also associated with some limitations such as (1) Limited efficacy in some types of cancer: The presence of a protective stromal barrier that blocks the virus from accessing the cancer cells has restricted the effectiveness of viro-immunotherapy in certain kinds of cancer. This may reduce the efficacy of viro-immunotherapy in some conditions. (2) Potential for immune-related adverse events: Viro-immunotherapy raises the risk of immunological-related side effects because it stimulates the immune system. Symptoms including the flu, inflammation, and autoimmune disorders are all potential side effects. (3) Pre-existing immunity to the virus: The therapeutic efficacy of the oncolytic virus may be reduced in patients who already have developed an immune response to the virus. This may occur as a result of previous contact with viruses or a tendency to mount an immune response against the virus as a result of a person’s genetic makeup. (4) Limited availability of clinical trials: Since viro-immunotherapy is still in its experimental stages, there are not many clinical trials available, that determine its safety and efficacy. Additionally, due to the novelty of viro-immunotherapy patients’ accessibility to this treatment option may be limited. (5) Potential for viral shedding: Viruses used in viro-immunotherapy can spread to other people especially patients with compromised immune systems. (6) Cost: Because of its complexity and high expense, viro-immunotherapy may be out of reach for many individuals. As indicated by the previous authors [119,121,136,137,138].

### 3.3. Cancer Vaccines

**Figure 8:**
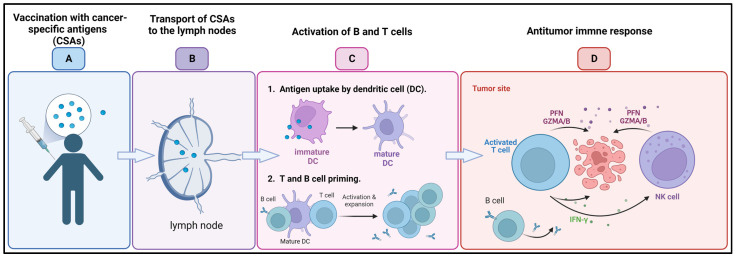
Cancer vaccine: (**A**) Following administration of cancer-specific antigens (CSAs) through vaccination (**B**) antigens are transported to the lymph nodes, where (**C**) they are uptaken by dendritic cells (DCs) and activate the B and T cells. (**D**) B cells produce tumor-targeted antibodies, while activated T cells and natural killer (NK) recruited to the tumor microenvironment release perforins (PFNs) and granzyme A/B (GZMA/B) and interferon γ (IFN-γ) leading to cancer cell death. Created with BioRender.com, accessed on 23 April 2023.

Cancer-preventive vaccines are intended to thwart the progression of cancer by either focusing on cancer-specific antigens that are expressed on precancerous or early-stage cancer cells or on viruses that are known to cause cancer [139]. On the other hand, therapeutic cancer vaccines (Figure 8) are intended to cure tumors that have already developed by enhancing the immune system’s capacity to recognize and destroy cancer cells. In recent decades, tremendous effort has been devoted to the development of cancer vaccines. One strategy that showed promise as a potential therapeutic option is the use of cancer-specific antigens (CSAs). CSAs are proteins or peptides that are exclusive to cancer cells and are identified by the immune system as being of a foreign origin. Several CSAs have been investigated, and they are now being incorporated into cancer vaccines to elicit an immune response against cancer cells [140,141].

Carcinoembryonic Antigen (CEA): CEA is a glycoprotein that is overexpressed in many malignancies, especially colorectal cancer. Clinical trials using a CEA-based vaccination for the treatment of several human cancers have been designed based on experimental results achieved using animal models. DCs- and recombinant virus-based vaccinations appear to be the most reliable among the several CEA-based cancer vaccines. Vaccination against CEA has been shown to induce a strong immune response, leading to a slower rate of tumor progression and improved survival among certain cancer patients. However, in most cases, vaccination has failed to eradicate the tumor, in part because of the detrimental impact exerted by the tumor microenvironment on the immune response [142,143].

Melanoma-associated antigen-A3 (MAGE-A3): MAGE-A3 is highly expressed in a variety of malignancies, including melanoma, non-small-cell lung cancer, and head and neck cancer. MAGE-A3 vaccination has been studied in multiple clinical studies for its safety, and potential to increase survival rates and trigger an immunological response in patients with the above-mentioned cancers [144,145,146,147]. The exact cellular function of this protein has not been revealed. Yang et al. suggested that MAGE protein expression may actively contribute to the formation of malignancies and increase tumor survival through inhibition of the tumor suppressor TP53 protein and decrease in cell apoptosis [148].

Mucin 1 (MUC1): Glycoprotein MUC1 is overexpressed in several cancers, such as breast, pancreatic, and lung cancers. The MUC1 vaccination has shown promise in boosting survival rates and eliciting immune responses in patients with pancreatic cancer and non-small-cell lung cancer in clinical studies [149,150].

New York esophageal squamous cell carcinoma-1 antigen (NY-ESO-1): Several kinds of cancer, such as melanoma, lung cancer, and ovarian cancer, express the antigen. Because of its capacity to trigger spontaneous humoral and cellular immune responses, as well as its restricted expression pattern, it is a promising candidate target for cancer immunotherapy [151].

Prostate-specific antigen (PSA): PSA is widely utilized in the identification and monitoring of prostate cancer. Vaccines based on PSA have been the subject of many clinical trials, some of which have shown encouraging results while others have been ambiguous [152,153,154].

Wilms Tumor 1 (WT1): WT1 is a transcription factor up-regulated in a wide variety of malignancies, including solid tumors, lymphomas, and leukemias. Patients with leukemia and mesothelioma who received WT1 vaccination in clinical trials experienced a significant increase in immune responses and survival rates [155,156].

Other include melanoma-associated antigen 1 (MZ2-E) and melanoma-associated antigen 3 (MZ2-D) from the melanoma-associated antigen gene family. They were effectively recognized by many immune cells, including cytotoxic T cells, and have been shown to elicit antitumor immune responses in melanoma-bearing cancer patients [157].

**Figure 9:**
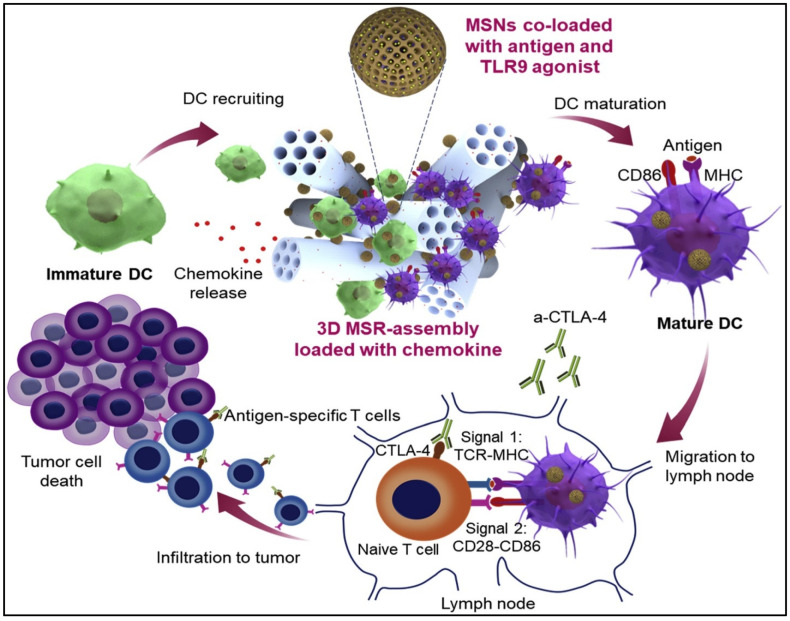
Efficient dendritic cell (DC) recruitment and activation by injectable dual-scale mesoporous silica microrod–mesoporous silica nanoparticle vaccine. Adapted with permission from Nguyen et al. [42].

Dendritic cell vaccines, which use DCs to trigger an immune response against cancer cells, are another potential treatment option. DCs are specialized immune cells capable of presenting antigens to T cells. DC vaccines have shown promise in clinical trials for a range of malignancies, including melanoma and prostate cancer [158]. Furthermore, several different kinds of macroporous scaffolds were created to release DC-recruiting chemokines, which would trigger the activation and maturation of these DCs. In a recent study, Nguyen et al. [42] developed an injectable dual-scale mesoporous silica vaccine enclosing chemokine, thereby facilitating the recruitment of numerous DCs into the scaffold composed of Toll-like receptor 9 (TLR-9) agonist and an antigen, which internalized by the recruited DCs became effectively presented [42]. The use of aerogels and mesoporous silica nanoparticles attracted tremendous attention in recent years [159,160]. Figure 9 presents efficient DC recruitment and activation by injectable dual-scale mesoporous silica microrod–mesoporous silica nanoparticle vaccine.

Exosomes are small vesicles that are connected to membranes and are produced by the majority of cells for intercellular exchange and regulation. These exosomes deliver materials or messages to the cells they are targeting for a variety of physiologically significant purposes. Exosomes that were derived from M1-polarized, proinflammatory macrophages were the subject of the research that was conducted by Cheng et al. The researchers were interested in the possibility of using M1 exosomes as an immunopotentiator for a cancer vaccine. After being injected subcutaneously, the M1 exosomes showed a tropism toward lymph nodes. They were predominantly uptaken by the local macrophages and DCs and triggered the secretion of a pool of Th1 cytokines. M1, but not M2, exosomes increased the activity of a lipid calcium phosphate (LCP) nanoparticle-encapsulated tyrosinase-related protein 2 (TRP2) vaccine and elicited a stronger antigen-specific cytotoxic T-cell response. In a melanoma growth inhibition study, M1 exosomes showed to be a more potent immunopotentiator than an adjuvant, such as CpG oligonucleotides (ODNs) when combined with LCP nanoparticle vaccine in a melanoma. This study demonstrated the potential of M1-polarized macrophage-derived exosomes as a vaccine adjuvant [161].

Despite the large number of vaccine strategies that are being developed regularly, the construction of safe and successful therapeutic cancer vaccines remains a challenge [140,162,163]. Advantages of their use include the prevention of cancer—vaccines against cancer-causing viruses including human papillomavirus (HPV) and hepatitis B virus (HBV) can be used to protect against the onset of cancer. Cancers such as cervical and liver cancer have been proven to decrease in frequency after vaccination against these viruses. Fewer side effects are another advantage due to cancer vaccines target only cancer cells, leaving healthy cells unharmed, making them safer than standard cancer therapies such as chemotherapy and radiation. On the other hand, the disadvantages include limited efficacy: not all individuals who receive a vaccine will experience a positive response. Cancer vaccines’ success rates may differ among patients with different cancer stages. High cost: cancer vaccines can be pricey, especially if they are customized for each patient, and may not be affordable for everyone. Additionally, development challenges: it can be difficult and time-consuming to develop a vaccine against cancer since accomplishing it requires finding the right target antigens, refining the vaccine’s formulation, and testing it in humans to make sure it is safe and effective.

### 3.4. Cytokine Therapies

Cytokines are chemical messengers secreted by immune and non-immune cells in reaction to cellular stresses such as bacterial infections, inflammation, and tumorigenesis to control cellular interactions [164]. The first use of cytokines in cancer treatment occurred in 1976, following the discovery of interleukin-2 (IL-2), initially known as a T-cell growth factor, which appeared to have the capacity to activate T cells and thus exert immune-stimulatory properties [164,165]. In vitro incubation of inactive lymphoid cells with recombinant IL-2 results in the production of lymphokine-activated killer (LAK) cells, which are cells that are capable to lyse tumor cells. The administration of LAKs and large doses of IL-2 in patients with various types of cancer have been reported to cause cancer regressions and significantly enhance antitumor response in preclinical studies and clinical trials [166,167].

More than half a century ago, type I interferons (IFNs), were identified for the first time as the factors responsible for virus inhibition. However, in the last ten years, researchers have only just started to understand the precise function that type I IFNs play in the body’s natural immune response to cancer. IFNs have been implicated in two primary functions in tumor control—induction of tumor cell senescence and stimulation of DC maturation to boost the T-cell cytotoxicity [168]. Cauwels et al. [169] developed a novel strategy for cancer treatment using Activity-on-Target cytokines, which are modified immune-cytokines that possess up to 1000-fold more potency for target cells. The authors reported that modified type I interferon designed to target Clec9A+ DCs was able to limit the progression of tumors and showed strong antitumor activity in breast carcinoma, murine melanoma, and lymphoma models, without toxic side effects. IFN activation of immune cells may inhibit tumor angiogenesis, whereas ILs promote the activation and growth of helper and cytotoxic T lymphocytes (CD4+ and CD8+ T cells) [170,171]. Granulocyte-macrophage colony-stimulating factor (GM-CSF) is another cytokine that, in reaction to stress, infections, and malignancies, stimulates the formation of myeloid cell subgroups including neutrophils, monocytes, macrophages, and DCs. GM-CSF has a global effect on the host immune surveillance system when pathologic conditions are present. When compared to other soluble immune mediators, it has been discovered that either an excess of or a deficiency in GM-CSF can encourage the aggressiveness of cancer. An insufficient amount of GM-CSF inhibits the proper production of innate immune cells, which in turn prevents the resulting induction of adaptive anticancer immune responses. On the other hand, too much GM-CSF can exhaust immune cells and encourage the growth of cancer [172,173]. GM-CSF has been also used to accelerate and augment granulocyte recovery after chemotherapy of cancers [174,175]. Cytokines such as INFγ or tumor necrosis factors (TNFs) are essential in the development of an immunogenic microenvironment. Anthracyclines as well as other treatments such as photodynamic therapy (PDT) exert their impacts on cancer cells in a way that stimulates the body’s immune system. This process, also known as immunogenic cell death (ICD), is distinguished by the release of membrane-bound and soluble factors that boost the function of immune cells [176] and can act as pro-inflammatory factors (TNFs, IL-1β, and IL-6) or can lead to increase in the expression of MHC class I on the surface of antigen-presenting cells. This leads to improved immune response via the differentiation promotion or the activation of both T cells and NK cells [177].

**Figure 10:**
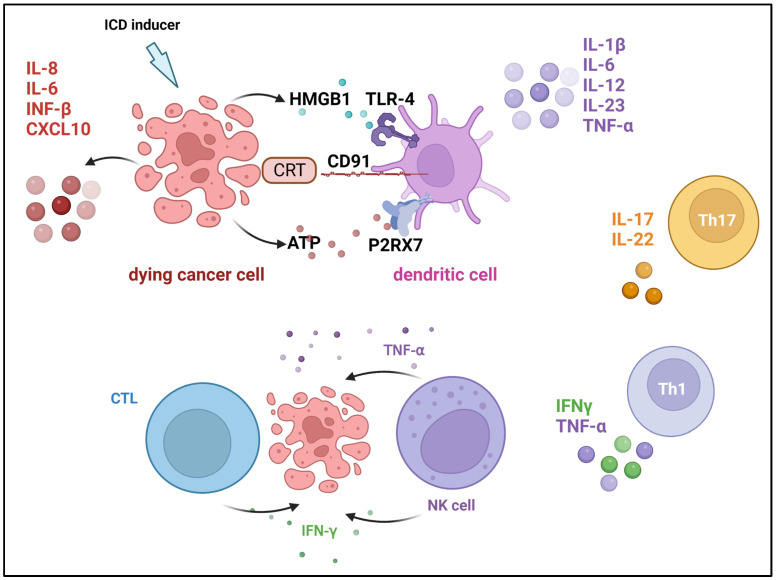
Cytokines that are involved in immunogenic cell death induction. Damage-associated molecular patterns (DAMPs) are released from dying cells during immunogenic cell death (ICD) and facilitate tumor antigen presentation and augment adaptive immunity. Cancer cells secrete calreticulin (CRT), which binds to the CD91 receptor on dendritic cells (DCs), and high-mobility group protein 1 (HMGB1), which is a ligand for the Toll-like receptor (TLR-4) on DCs. In addition, DSc’s P2X purinoceptor 7 (P2RX7) engages with ATP that cancer cells secrete. Created with BioRender.com, accessed on 23 April 2023. Modified from Showalter et al. [176].

Immunogenic cell death inducers trigger the release of damage-associated molecular patterns (DAMPs) such as high mobility group box protein 1 (HMGBP1), CRT, and ATP, as well as inflammatory cytokines from cancer cells. Both DAMPs and cytokines send activating signals for DCs and NK cells, which respond instantly by releasing effector cytokines [178]. Dying tumor cells treated with ICD inducers trigger the release of various cytokines, which modulate an effective immune response, such as IL-6 and IL-8, [179]. NK cells functionality appears to be increased by cytokines released from activated DCs, such as IL-12, as well as cytokines produced by other innate immune cells, such as IFN-α/β, leading to the secretion of IFN-γ and TNFα (Figure 10) [177].

**Figure 11:**
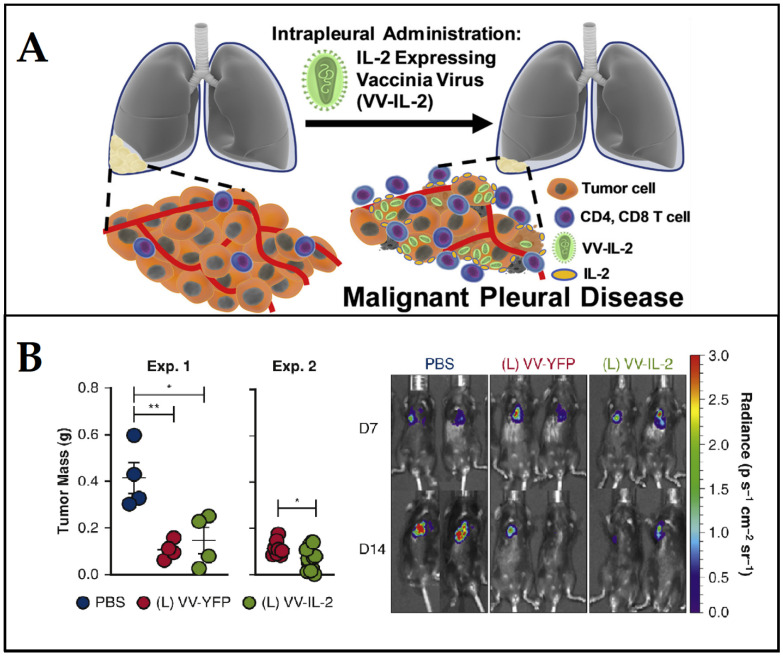
Antitumor effect of vaccinia virus expressing interleukin-2 (VV-IL-2) against malignant pleural disease. (**A**) The principal of the approach; (**B**) antitumor effect upon the administration of VV-IL-2, which reduced tumor burden and increased PD-1+ tumor-infiltrating T lymphocytes and survival. Phosphate-buffered saline (PBS) was used as a control (* *p* < 0.05, ** *p* < 0.01). Adapted from Ekeke et al. [180].

Ekeke et al. [180] used the vaccinia virus to up-regulate the expression of IL-2 to activate antitumor response against malignant pleural disease and to trigger the immune modulatory effects. Local administration of an IL-2-expressing vaccinia virus (VV-IL-2) significantly reduced tumor burden, due to the increase in the activity of immune cells including CD4+ and CD8+ T cells and α,β T-cell receptor diversity in a malignant pleural disease model (Figure 11).

While cytokine treatments have the potential to improve cancer treatment, they also pose considerable risks [181,182,183,184,185,186]. Below are the pros and negatives of their use:

1. Stimulation of the immune system: the immune system can be stimulated with cytokine treatments to better target and destroy cancer cells.
2. Reduced toxicity: compared to conventional treatments such as chemotherapy and radiation, cytokine therapies are safer and have fewer adverse effects.
3. Versatility: cytokine treatments are adaptable because they can be used to treat a wide variety of cancers.
4. May provide long-term benefits: some cytokine therapies have been demonstrated to produce lasting advantages in cancer patients, increasing the likelihood of a long-lasting response to treatment.
5. Can be used in combination with other treatments: combining cytokine therapies with standard cancer treatments such as chemotherapy, radiation therapy, and immunotherapy has been shown to improve patient outcomes.
6. May induce remission: some cytokine therapies, such as high-dose IL-2 and IFN-α, have been shown to induce complete or partial remission in some cancer patients.
7. Long-term immune memory: long-term immunological memory can be induced by some cytokine therapies, such as IL-2, meaning that the immune system will continue to detect and fight cancer cells even after treatment has ended.
8. Fewer treatment sessions: patients may find cytokine therapies more convenient because they often need fewer treatment sessions than treatments such as chemotherapy.

Although cytokine treatments have shown promise in the treatment of cancer, further study is needed to determine their full potential. It is important to overcome the potential limitations of this therapeutic approach:

1. Limited effectiveness: some patients may only have a partial response to cytokine treatments, and they may not work for all forms of cancer.
2. Side effects: side effects from cytokine therapies include fever, exhaustion, and muscle aches, but they are typically less severe than those from chemotherapy and radiation. Some cytokine therapies, such as high-dose IL-2, can cause damage to organs such as the kidneys and liver, which can be life-threatening.
3. Expensive: some patients may not be able to afford cytokine therapy due to their high cost.
4. Can cause autoimmune reactions: there are risks associated with cytokine treatments, including autoimmune reactions and tissue damage.

### 3.5. Adoptive Cell Transfer

**Figure 12:**
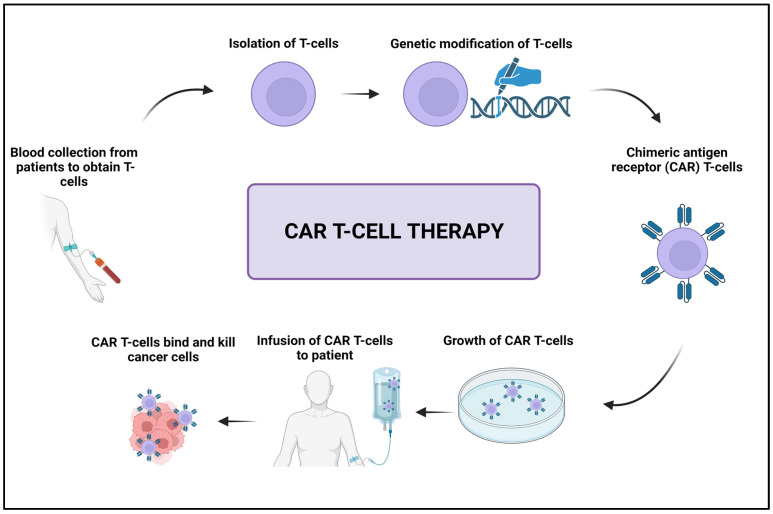
Schematic illustration of the CAR T-cell therapy. Created with BioRender.com, accessed on 23 April 2023.

Adoptive cell transfer (ACT) is a cancer treatment method that involves injecting immune cells, such as T cells, into a patient’s body, where they seek out and destroy cancer cells. Before being administered to a patient, T cells are usually modified or enhanced in the lab to strengthen their ability to recognize and target cancer cells. CAR T-cell therapy is a very successful form of ACT that involves engineering T cells to express chimeric antigen receptors (CARs) that can recognize and adhere to specific proteins on the surface of cancer cells (Figure 12) [187,188].

**Figure 13:**
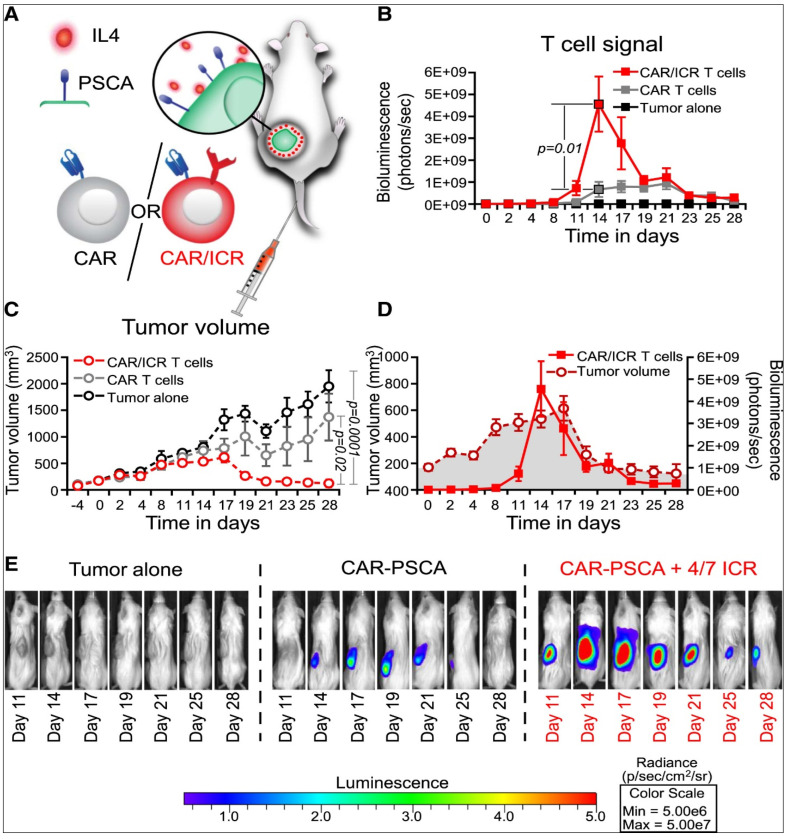
Inverted cytokine receptor-modified chimeric antigen receptor T cells exhibit noticeably antitumor activity. (**A**) Schematic drawing of mice engrafted with cancer cells and treated with either CAR/ICR T cells or CAR. (**B**) CAR and CAR/ICR T cells expansion and persistence as measured by bioluminescence imaging. (**C**) The reduction in tumor volume for CAR/ICR T cell-based treatment. (**D**) Superimposition of tumor volume and T-cell signal in CAR/ICR-based treated mice. (**E**) Images of mice administered with two treatments: CAR, CAR/ICR T cells, or control. Adapted with permission from Mohammed et al. [192].

Rosenberg et al. [166] revealed that the administration of modified autologous LAK cells in addition to recombination-derived IL-2 was effective in the treatment of patients with metastatic cancers. High response rates in patients with follicular lymphoma and diffuse large B-cell lymphoma have been reported with modified T cell-based therapy, using CAR able to target CD19 receptors on the surface of B-cell cancers [189]. Schuster et al. [190] used autologous modified T cells expressing a CD19-directed CAR to treat patients with follicular lymphoma or diffuse large B-cell lymphoma. The authors revealed that the engineered cells were effective in the treatment. High rates of durable disease reduction were observed, with significant recovery of B cells and immunoglobulins in some patients. Similarly, Wang et al. [191] conducted a trail on seven patients suffering from advanced diffuse large B cell lymphomas and concluded that anti-CD20 CAR T cells caused prolonged tumor regression. In a different study, Mohammed et al. [192] generated chimeric antigen receptor T cells directed against a specific antigen located on prostate stem cells and revealed specific tumor lysis. The authors generated an inverted receptor for the cytokine to protect the modified cells from the immunosuppressive cytokines, resulting in significant enhancement in antitumor activity. For the determination of whether the superior modified T-cell expansion and antitumor effects of chimeric antigen receptor/inverted cytokine receptor (CAR/ICR) could be recapitulated in vivo, the authors used mice models as presented in Figure 13, and treated animals intravenously with CAR/ICR T cells. Superior antitumor effects were observed with CAR/ICR T cells-based treatment, resulting in 100% overall survival for the treated group compared to the control group [192].

Similarly to the previously discussed immunotherapeutic approaches CAR T-therapies possess significant advantages over conventional chemotherapy and radiotherapy. In contrast to conventional chemotherapy, which is harmful to normal cells as well as cancerous ones, CAR T-cell therapy is a targeted technique that can selectively destroy cancer cells while sparing healthy ones. This lowers the toxicity and adverse effects of CAR T-cell treatment. CAR T cells may also persist in the body for an extended time and continue to attack cancer cells, offering protection against the disease even after the cells have been eliminated. The elimination of cancer cells using CAR T-cell therapy is curative in many patients. However, similar to other novel immunotherapies, the CAR T-cell approach is an expensive treatment. This can be attributed to the sophisticated manufacturing procedure for CAR T cells that can cause treatment delays for some patients. Moreover, it is crucial to determine which patients could benefit the most form the treatment and those with increased risk of detrimental side effects such as cytokine release syndrome (CRS) and immune effector cell-associated neurotoxicity syndrome (ICANS) [193,194].

### 3.6. Other Immune Therapies

Lag-3 (Lymphocyte Activation Gene 3) is a protein receptor expressed on the surface of certain immune cells, including T cells, B cells, and natural killer (NK) cells. Lag-3 plays a role in regulating immune responses and is considered an immune-checkpoint protein [195]. In the context of immunotherapy, Lag-3 has gained attention as a potential target for cancer treatment. Immune-checkpoint inhibitors, such as antibodies targeting PD-1 (programmed cell death protein 1) and CTLA-4 (cytotoxic T-lymphocyte-associated protein 4), have shown success in unleashing the immune system against cancer cells. However, not all patients respond to these therapies, highlighting the need for additional targets [196]. Several clinical trials are underway to investigate the efficacy of Lag-3 inhibitors either as monotherapy or in combination with other immunotherapies. Preliminary data from these trials suggest that Lag-3 inhibitors may provide clinical benefit in certain types of cancers, such as melanoma, lung cancer, and renal cell carcinoma [197,198]. It is important to note that the field of Lag-3 and immunotherapy is still evolving, and more research is needed to fully understand its role and potential in cancer treatment. Clinical trials will continue to provide valuable insights into the efficacy and safety of Lag-3-targeted therapies, as well as their optimal use in different cancer types and patient populations.

Nanoparticles and biomaterials have been employed to enable programmed localization, improve pharmacokinetics, and co-deliver immune-modulatory substances that were not previously possible with direct administration of these compounds in solution [199]. In a recent investigation, Ji et al. [200] designed a biopolymer immune implant for post-surgical therapy of colorectal cancer and revealed that an immune implant was able to eradicate residual tumors post-surgery for more than 150 days. The gradual release of the loaded resiquimod (Toll-like receptor 7/8 (TLR7/TLR8) agonist) was achieved and the anti-CD134 (OX-40) antibody elicited immune memory and inhibited the growth of distal tumors when used in the implant [200]. In previous studies, the OX40 and OX40 ligand interaction was shown to promote the growth and division of T cells while attenuating the immunosuppression of Treg cells. The OX40 plays a crucial part in immunity, hence it is the subject of numerous clinical trials to assess its therapeutic effects in the treatment of cancer [201]. Immunological studies demonstrated that treating patients with a biopolymer immune implant has a two-phase effect: initially, a rise in the infiltration of NK cells and DCs is observed in the first few days, followed by an increase in the infiltration of T cells and the establishment of immune memory in the following weeks [200]. Peptide-based materials constitute another approach to cancer immunotherapies that have been used to solve several traditional treatment challenges and have demonstrated specific and considerable efficacy as cancer immunotherapies [202]. Peptide-based cancer vaccines and delivery systems have been the subject of extensive research. These vaccines and systems aim to replicate the functional domains of proteins with highly specialized immuno-regulatory capabilities [202,203]. Shen et al. [204] reported a mono-palmitoylated peptide able to induce an anticancer immune response without any addition of an adjuvant. The authors also developed a long peptide of a di-palmitic acid conjugated with TLR2 agonist and achieved significant improvement in antitumor immunity by diminishing the function of tumor-associated macrophages (TAMs). Using a different approach, Choi et al. [205] investigated different peptide antigens from cancer stem cells to compose DC vaccination for hepatocellular carcinoma and human breast cancer. By pulsing dendritic cells with CD44 and epithelial cell adhesion molecule-based peptides, the authors reported an effective stimulation and mature DC production in addition to enhanced T-cell stimulation and significant increase in the number of CTLs [205].

## 4. Challenges and Prospects of Cancer Immunotherapy

Improving the immune response against cancer can be accomplished by suppressing or activating immunological checkpoint proteins on T cells. Anti-CTLA-4 and anti-PD-1/PD-L1 checkpoint inhibitors have shown promise in the treatment of melanoma, non-small-cell lung cancer, and bladder cancer. These drugs, however, have been linked to immune-related side effects such as colitis, hepatitis, and pneumonitis [93,206]. The goal of oncolytic viral therapy is to stimulate an immune response against the tumor by selectively infecting and destroying cancer cells. These treatments have shown promise in studies on a variety of cancers, including melanoma, glioblastoma, and pancreatic cancer. However, because of difficulties with virus transmission to tumor locations and preexisting immunity, they may not be as effective as intended [119,121,136,137,138,207]. Cancer vaccines work by inducing an immune response against cancer-causing antigens to prevent or treat the disease. Cancer vaccines that have been developed and tested include peptide vaccines, DC vaccines, and whole-cell vaccinations. Despite encouraging achievements in generating an immune response, most vaccinations still have low clinical effectiveness. Some issues with the use of cancer therapeutic vaccines to treat various types of malignancies have been described, such as the nonresponsiveness of activated T cells due to reduced antigen presentation and/or increased suppression of self-reactivity by Tregs [140,141,208]. Another concern with therapeutic vaccines is that they are inadequately immunogenic, resulting in self-derived antigens that limit the immune response. However, certain attempts to boost such responses have resulted in the development of autoimmune reactions in patients [209,210]. Cytokine treatments include the injection of patients with cytokines, which are signaling molecules produced by immune cells, to improve the immune system’s defenses against cancer. However, these treatments can have serious adverse effects and are only partially successful against certain types of cancer [184]. T cells are extracted from a patients, grown and manipulated to attack cancer-related antigens, after their reintroduction to the patient. CAR- T-cell therapy, which involves engineering T cells to express CARs that identify cancer cells, has shown promising results in hematological malignancies such as leukemia and lymphoma. Although CAR- T-cell therapy has shown promise, its success in treating solid tumors is currently limited. Any of these strategies, used alone or in combination, can help to boost the immunological response against cancer [211,212].

The kind, stage, and location of cancer, as well as the patient’s overall health and immune status, are all important factors to consider when deciding on an immunotherapeutic strategy. The inability to anticipate the clinical efficacy of some novel techniques, such as the use of immunotherapy combinations, must still be evaluated at various phases of clinical studies. Many patients obtain considerable remission and prolonged survival after receiving cancer immunotherapies, but the long-term implications of these treatments are still being investigated [190,213]. The optimization of treatment duration, therapeutic dose or schedules and establishment of novel surrogate endpoints that specifically capture the overall survival benefit of treatment are needed [214]. To ensure the safety of these breakthrough therapies, specific biomarkers capable of providing evidence-based decisions for the success of immunotherapy need to be established [215,216].

## 5. Conclusions

The past two decades have witnessed a significant understanding of the role of the immune system in the control of malignancies as well as methods employed by cancer cells to avoid immunosurveillance. Cancer immunotherapy has shown remarkable success in treating various types of cancer, including melanoma, lung cancer, and bladder cancer, and certain types of leukemia and lymphoma. Many types of cancer immunotherapy have been developed including immune-checkpoint inhibitors, oncolytic viro-therapies, cancer vaccines, cytokine therapies, and adoptive cell transfer technology, with significant clinical improvements in patients’ survival and quality of life. This has revolutionized cancer treatment and has the potential to provide long-term remission or even cure in some cases. However, it is important to note that not all patients respond to immunotherapy, and further research is ongoing to improve its effectiveness and identify biomarkers that can predict response to treatment. More investigations on long-term effects and the possibility of developing autoimmune illnesses are needed to ensure the safety of these therapies. The next generation of cancer immunotherapies will transform the battle against cancer, making the disease preventable, controllable, or even curable.

## Figures and Tables

**Table 1:** Summary of the chronological events of cancer immunotherapy.

Scientist/s and Year	Remark	Ref
W. B. Coley in 1893	The first use of immunotherapy to treat a cancer patient.	[45]
Hericourt and Richet in 1895	Serotherapy in cancer treatment.	[47]
W. B. Coley in 1899	The first vaccine against sarcoma.	[48]
Leyden et al. in 1902	The first cancer vaccine was created using a patient’s tumor cells.	[49]
J. B. Murphy in 1914	Understanding the function of lymphocytes in the rejection of grafted tumors.	[50]
Ernest Witebsky in 1929	Tumor molecular markers are used as antigens.	[51]
Burnet and Lewis in 1957	The concept of the natural anticancer immune response.	[52]
Sjögren et al. in 1971	The role of antibodies in shielding tumor cells from the anticancer immune reaction.	[53]
Carswell et al. in 1975	Discovery of tumor necrosis factor (TNF-α) with antitumor effects.	[54]
Morgan et al. in 1976	The first discovery of IL-2 as a T-cell growth factor.	[55]
Stevenson and Elliott in 1977	First anti-idiotype antibodies activate immune cells against the tumor.	[56]
Ronald Levy in 1981	First successful cancer treatment with anti-idiotype antibody.	[57]
Taniguchi et al. in 1983	First cloning of T-cell growth factor (IL-2).	[58]
Kappler et al. in 1983	First identification of T-cell antigen receptor.	[59]
Knuth et al. in 1984	First clinical trial on the potential use of T cells to fight off tumors.	[60]
S A Rosenberg in 1988	Successful application of IL-2-based cancer therapy.	[61]
Gross et al. in 1989	The first use of genetically engineered T cells for tumor targeting.	[62]
Dranoff et al. in 1993	Using macrophage colony-stimulating factor (M-CSF) to improve cancer immunity.	[63]
Leach et al. in 1996	Inhibition of the immune cells CTLA-4 molecule in cancer treatment.	[64]
Levy and Rastetter in 1997	First FDA-approved monoclonal antibody as an anticancer drug.	[65]
J. P. Allison in 2000	First immune-checkpoint inhibitor drug against CTLA-4.	[66]
Hirano et al. in 2005	Development of fully human anti-PD-1 antibody for cancer therapy.	[67]
Kershaw et al. in 2006	Development of adoptive cell-mediated immunotherapy.	[68]
Rosenberg et al. in 2008	Anti-PD-1 antibody started phase 1 of the clinical trial for cancer	[69]
June and Whitehead in 2012	First leukemia treatment by adoptive cell (CAR-T based therapy).	[70]
Quezada et al. in 2016	Genetic modification of immune cells to prompt antitumor response.	[71]
Wallace et al. in 2016	A first clinical trial of gene editing tool CRISPR/Cas 9 for cancer.	[72]
Freedman et al. in 2018	Genetically engineered virus to selectively kill cancer cells.	[73]
Zhang et al. in 2020	The use of miRNA to regulate immune checkpoints.	[74]

**Table 2:** Important and commonly used oncolytic viruses in cancer immunotherapy.

Virus (*Family*)	Nucleic Acid Type (Group)	Study Remarks	Ref
Reovirus (*Reoviridae*)	dsRNA (G III)	Viruses exhibited a safe and tolerable toxicity profile in early clinical trials, with minimal viral shedding, replication, localization, and cytotoxic effects.	[122]
Influenza A Virus (*Orthomyxoviridae*)	ssRNA-ve (G V)	Engineered viruses exhibited tumor-ablative potential and do not replicate in normal cell lines.	[123]
Herpes Simplex Virus1 (*Herpesviridae*)	dsDNA (G I)	A virus exhibited high antitumor efficacy, has a high safety margin, is well tolerated and its use is associated with a low frequency of adverse effects in most of the treated patients.	[124]
Adenovirus (*Adenoviridae*)	dsDNA (G I)	Clinical data revealed that the virus was tolerable and effective even when combined with other therapeutic approaches.	[125]
Measles virus (*Paramyxoviridae*)	ssRNA-ve (G V)	The virus replicated selectively in the tumor and significantly suppressed its development. It was completely safe and did not result in any measles-like symptoms.	[126]
Vaccinia virus (*Poxviridae*)	dsDNA (G I)	The virus replicated rapidly in tumor cells with significant antitumor effects, but its cytotoxicity varied based on cell lines.	[127]
Pseudorabies (*Herpesviridae*)	dsDNA (G I)	Selective replication of viruses has been observed in many kinds of cancers, with tumor growth being significantly slowed by more than 50%.	[128]

## Data Availability

Not applicable.
